# Patients' and Nurses’ Experiences and Perceptions of Remote Monitoring of Implantable Cardiac Defibrillators in Heart Failure: Cross-Sectional, Descriptive, Mixed Methods Study

**DOI:** 10.2196/19550

**Published:** 2020-09-28

**Authors:** Maria Liljeroos, Ingela Thylén, Anna Strömberg

**Affiliations:** 1 Department of Health, Medicine and Caring Sciences Linköping University Linköping Sweden; 2 Centre for Clinical Research Sörmland Uppsala University Eskilstuna Sweden; 3 Department of Cardiology Linköping University Linköping Sweden

**Keywords:** heart failure, remote patient monitoring, implantable cardioverter-defibrillator

## Abstract

**Background:**

The new generation of implantable cardioverter-defibrillators (ICDs) supports wireless technology, which enables remote patient monitoring (RPM) of the device. In Sweden, it is mainly registered nurses with advanced education and training in ICD devices who handle the arrhythmias and technical issues of the remote transmissions. Previous studies have largely focused on the perceptions of physicians, and it has not been explored how the patients’ and nurses’ experiences of RPM correspond to each other.

**Objective:**

Our objective is to describe, explore, and compare the experiences and perceptions, concerning RPM of ICD, of patients with heart failure (HF) and nurses performing ICD follow-up.

**Methods:**

This study has a cross-sectional, descriptive, mixed methods design. All patients with HF and an ICD with RPM from one region in Sweden, who had transitioned from office-based visits to implementing RPM, and ICD nurses from all ICD clinics in Sweden were invited to complete a purpose-designed, 8-item questionnaire to assess experiences of RPM. The questionnaire started with a neutral question: “What are your experiences of RPM in general?” This was followed by one positive subscale with three questions (score range 3-12), with higher scores reflecting more positive experiences, and one negative subscale with three questions (score range 3-12), with lower scores reflecting more negative experiences. One open-ended question was analyzed with qualitative content analysis.

**Results:**

The sample consisted of 175 patients (response rate 98.9%) and 30 ICD nurses (response rate 60%). The majority of patients (154/175, 88.0%) and nurses (23/30, 77%) experienced RPM as very good; however, the nurses noted more downsides than did the patients. The mean scores of the negative experiences subscale were 11.5 (SD 1.1) for the patients and 10.7 (SD 0.9) for the nurses (*P*=.08). The mean scores of the positive experiences subscale were 11.1 (SD 1.6) for the patients and 8.5 (SD 1.9) for the nurses (*P*=.04). A total of 11 out of 175 patients (6.3%) were worried or anxious about what the RPM entailed, while 15 out of 30 nurses (50%) felt distressed by the responsibility that accompanied their work with RPM (*P*=.04). Patients found that RPM increased their own (173/175, 98.9%) and their relatives’ (169/175, 96.6%) security, and all nurses (30/30, 100%) answered that they found RPM to be necessary from a safety perspective. Most patients found it to be an advantage with fewer office-based visits. Nurses found it difficult to handle different systems with different platforms, especially for smaller clinics with few patients. Another difficulty was to set the correct number of alarms for the individual patient. This caused a high number of transmissions and a risk to miss important information.

**Conclusions:**

Both patients and nurses found that RPM increased assurance, reliance, and safety. Few patients were anxious about what the RPM entailed, while about half of the nurses felt distressed by the responsibility that accompanied their work with RPM. To increase nurses’ sense of security, it seems important to adjust organizational routines and reimbursement systems and to balance the workload.

## Introduction

According to clinical guidelines, an implantable cardioverter-defibrillator (ICD), with or without cardiac resynchronization therapy (CRT), is recommended in selected patients with heart failure (HF) to reduce the risk of sudden cardiac death. CRT uses a special type of pacemaker called a biventricular pacemaker to treat HF. The CRT pacemaker is placed under the skin of the chest and connects to three leads in the heart: one in the right atrium, one in the right ventricle, and one in the left ventricle. The pacemaker sends electrical pulses to make the ventricles pump at the same time. The pacemaker can also speed up the heartbeats if the heart is beating too slowly. When the lead in the right ventricle is combined with a shock lead, the device is called an ICD, which continuously monitors the heart rhythm and, in case of abnormal ventricular rhythm, can shock the heart back to a normal rhythm. The indication could be either secondary (ie, in patients recovering from ventricular arrhythmia causing hemodynamic instability) or primary (ie, in patients who are at high risk for life-threatening ventricular arrhythmias in the future: primary prevention) [1]. The number of patients treated with a primary ICD is increasing and account for about 80% of all implants worldwide today [2].

Traditionally, ICD follow-ups have required in-person assessment, with quarterly to yearly office-based visits and with an increased rate when the device approaches its end of service, in case of advisories, or when the patient’s health deteriorates. During these follow-up visits, a cardiologist, a registered nurse specialized in the care of ICD patients, and/or a technician noninvasively uses a programmer to gather data from the device. However, in the last decade the new generation of ICD devices that supports wireless technology has enabled remote monitoring of the device [3-6]. Consequently, in order to improve clinical practice of ICD follow-ups and to provide earlier detection of clinical problems, a wider use of remote patient monitoring (RPM) has been recommended by scientific associations in both Europe [5] and the United States [7], and has become the new standard of care for patient follow-up [8-10]. When a patient is remotely followed, the office-based visits can be limited to the initial postimplantation period and annual follow-ups, unless alerts or the patient’s health requires an urgent in-person check [6]. In practice, the data transceiver is typically situated in the patient’s bedroom to automatically receive data from the implant, using wireless Bluetooth technology, during the night, usually between 1 AM and 5 AM. In case of an alert (ie, arrhythmia or technical problem), the data are automatically transmitted to the manufacturer’s central repository using a mobile network link or a landline where the personnel at the device clinic have access to the data on a secure, dedicated website.

Remote monitoring of implanted devices has been found to have a number of advantages for both patients and health care personnel, compared to office-based visits, when the connectivity and transmissions work properly [11]. For example, RPM of the ICD reduces health care costs, time consumption, as well as transportation costs for patients [12,13]. It also provides a feeling of security for the patients knowing that their device is constantly being monitored and if there are some functional problems, those will be detected without delay [14]. Previous studies also indicate that ICD patients are generally satisfied with RPM [15,16], and comprehension of the usefulness of RPM has been positively associated with the acceptance of being monitored remotely [15,16]. Physicians also regard RPM as a clinically useful technology that affords significant benefits for patients and health care organizations, with the most significant benefit being the early detection of atrial arrhythmias, lead failure, and worsening of HF in CRT patients [17]. The EVOLVO (Evolution of Management Strategies of Heart Failure Patients With Implantable Defibrillators) study demonstrated a reduction of 35% in urgent admissions and 21% in urgent office-based visits for worsening HF in the RPM arm, even though this study was not powered to demonstrate clinical benefit [18]. Further, the REMOTE-CIED ( Remote patient management for Cardiac Implantable Electronic Devices) trial showed that patient-reported health status and ICD acceptance did not differ between patients on RPM and patients receiving in-clinic check-ups alone in the first 2 years after ICD implantation [19].

However, disadvantages have also been described. For example, some patients (5%-22%) do not feel comfortable with RPM and report a strong preference for regular office-based visits to feel secure [15,16]. Furthermore, RPM has been described as system centered, providing patients with little or no data from their device [20]. Patients missed receiving feedback via their monitor, 84% wished for a more detailed response, and 21% wished for faster feedback after scheduled transmissions [16,21]. Yet another disadvantage is the lack of data integration with electronic medical record platforms and other systems in the current RPM systems, leading to the device diagnostics being underutilized by the health care personnel [20]. Despite a reduction of office-based follow-ups, RPM is perceived as increasing workload for the staff involved [17]. Furthermore, the American PREDICT-RM (Patient-Related Determinants of ICD Remote Monitoring) registry has also found that about every fourth patient chose not to activate their RPM system at home, leading to extra time consumption for the staff in identifying and contacting those patients. Younger age, racial and ethnic minorities, having no health insurance, shorter travel distance to the hospital, and the presence of comorbidities or procedure-related adverse events have been found to be associated with a lower likelihood of RPM activation [22]. Finally, the EDUCAT study showed that a high overall understanding of RPM was related to patient age, where younger patients had a better comprehension of home monitoring but the number of data transmissions were unrelated to comprehension, which confirms the importance of training in patients’ acceptance of the system [23].

In Europe with its different health care and reimbursement systems, the heterogeneity of follow-up appointments is quite substantial when it comes to the accumulative time spent and the number of health care personnel involved in the visits, as described in a survey by the European Heart Rhythm Association in 2011. This *snapshot* survey included 26 *real-word practice* centers from seven European countries—the United Kingdom, Spain, Switzerland, France, Germany, Italy, and Greece—and showed that the mean duration of a visit was 27 minutes (25^th^ and 75^th^ percentiles were 15 and 35) for scheduled office-based visits and that most visits involved a cardiologist and a nurse simultaneously (59%). Nurses alone did 4% of the face-to-face visits [24]. However, in Sweden, it is mainly registered nurses with advanced education and training in ICD devices who handle the arrhythmias and technical issues of the ICDs and who consult a cardiologist when needed. There is no uniform education and training on ICD monitoring. Some of the manufacturers offer product-specific education online repeatedly every year; others offer short written instructions. It is usually up to the different clinics to educate and train new nurses by introducing them to the task assignment. Previous studies have largely focused on the perceptions of physicians, and it has not been explored how the patients’ and nurses’ experiences and perceptions of RPM compare to each other. Since nurses worldwide have become more involved in the care for ICD patients with RPM, and as the number of patients with RPM of their devices increases, it is important to explore their experiences.

Nurses in Sweden usually work with one platform per manufacturer, and each nurse has to handle three to four different platforms, each covering about five to ten different ICD-CRT-D (cardiac resynchronization therapy defibrillator) models or systems, depending on the procurement in the respective region.

Therefore, the aim of the study was to describe, explore, and compare experiences and perceptions of cardiac nurses performing ICD follow-up, concerning RPM of ICD in patients with HF.

## Methods

### Design and Setting

This study had a cross-sectional, descriptive, mixed methods design. All patients with HF and an ICD with RPM from one region in Sweden were invited to participate in the study. In addition, all ICD nurses working at all the ICD clinics in Sweden were invited to complete a survey.

The region of Sörmland has a land area of 6103 km^2^ and about 300,000 inhabitants. There are three hospitals with a total of 270 hospital beds on medical wards, but only one hospital has an in-hospital device clinic. The travel distance for patients with an implanted device could be up to 100 km.

Over 1 year, from October 2015 until October 2016, RPM was introduced and started for all ICD recipients during their scheduled visits at the device clinic, and all new ICD recipients received RPM directly after the implant. Due to various reasons, 4 patients declined RPM, so at the end of 2018 there were 310 ICD recipients on RPM in Sörmland County.

Before implementing RPM, ICD recipients had scheduled office-based visits every 3 to 6 months. After the implementation of RPM, patients routinely visit the clinic once a year and, in between, a scheduled transmission is performed. The ICD recipients can call the ICD nurse on weekdays when needed.

### Sample and Procedure

All adult ICD recipients having a verified HF diagnosis according to the European Society of Cardiology guidelines [1] (N=177) were invited to participate in the study during their yearly follow-up visit at the in-hospital device clinic, from January to December 2018. Exclusion criteria were being less than 18 years old and not being able to understand Swedish.

Patients interested in participating provided written informed consent. They were thereafter given additional written information and questionnaire packets to complete at home and return by mail in prepaid envelopes.

The ICD nurses were identified by contacting the National Swedish Pacemaker and ICD Registry, which provided names and email addresses for all ICD nurses (N=50) working at an ICD clinic in Sweden at the time. An electronic survey was sent out and two reminders were posted.

### Measures and Instruments

#### Overview

Demographic data and data on comorbidities were self-reported by the patients. These data included gender, age, living arrangements, place of birth, and educational level. The patients also self-reported validated measures concerning the level of ICD-related concerns [25], symptoms of depression and anxiety [26-28], and perceived control [29,30]. Demographic data from the nurses were also self-reported and included gender, age, work experience, number of patients at the clinic, number of patients on RPM, and time spent per week working with RPM.

#### Experiences of Remote Monitoring

Two 8-item questionnaires to assess patients’ and nurses’ experiences of remote monitoring were developed by the study team (see Table 1). Patients rated the items (eg, “RPM is technically difficult for me”) on a 4-point Likert scale from 1 (Totally agree) to 4 (Do not agree).

The nurses answered similar questions on a 4-point Likert scale, but their questions also concerned how comfortable they were handling RPM and the responsibility this brings (eg, “The responsibility that accompanies my work with RPM worries me” and “The responsibility that accompanies my work with RPM increases my security”).

For both patients and nurses, there was one final open-ended question: “I experience these advantages or disadvantages of RPM”; it was possible to write as many comments as one liked when answering the question.

**Table 1:** Questionnaire items and responses to assess patients’ and nurses’ experiences of remote patient monitoring (RPM).

Questions or statements		Responses
**Patient questionnaire**		
	1. What are your experiences of RPM in general?	1 (Bad), 2 (Fairly bad), 3 (Fairly good), or 4 (Good)
	2. RPM is unnecessary.	1 (Totally agree), 2 (Mostly agree), 3 (Partly agree), or 4 (Do not agree)
	3. RPM is technically difficult for me.	1 (Totally agree), 2 (Mostly agree), 3 (Partly agree), or 4 (Do not agree)
	4. RPM makes me worried.	1 (Totally agree), 2 (Mostly agree), 3 (Partly agree), or 4 (Do not agree)
	5. RPM increases my security.	4 (Totally agree), 3 (Mostly agree), 2 (Partly agree), 1 (Do not agree)
	6. RPM makes me feel safe.	4 (Totally agree), 3 (Mostly agree), 2 (Partly agree), 1 (Do not agree)
	7. RPM provides increased security and safety for my relatives.	4 (Totally agree), 3 (Mostly agree), 2 (Partly agree), 1 (Do not agree)
	8. I experience these advantages or disadvantages of RPM.	Open-ended question
**Nurse questionnaire**		
	1. What are your experiences of RPM in general?	1 (Bad), 2 (Fairly bad), 3 (Fairly good), or 4 (Good)
	2. RPM is unnecessary.	1 (Totally agree), 2 (Mostly agree), 3 (Partly agree), or 4 (Do not agree)
	3. RPM is technically difficult for my patients.	1 (Totally agree), 2 (Mostly agree), 3 (Partly agree), or 4 (Do not agree)
	4. The responsibility that accompanies my work with RPM worries me.	1 (Totally agree), 2 (Mostly agree), 3 (Partly agree), or 4 (Do not agree)
	5. The responsibility that accompanies my work with RPM increases my security.	4 (Totally agree), 3 (Mostly agree), 2 (Partly agree), 1 (Do not agree)
	6. RPM is necessary from a patient safety perspective.	4 (Totally agree), 3 (Mostly agree), 2 (Partly agree), 1 (Do not agree)
	7. RPM gives the patient increased security and safety.	4 (Totally agree), 3 (Mostly agree), 2 (Partly agree), 1 (Do not agree)
	8. I experience these advantages or disadvantages of RPM.	Open-ended question

### Data Analysis

Descriptive statistics were used to present sample characteristics for all study variables. Demographic and clinical variables were compared using chi-square statistics or the Student *t* test.

Regarding experiences of remote monitoring, for both patients and nurses, the items were divided into two subscales based on an exploratory factor analysis: one with negative experiences (items 2-4) and one with positive experiences (items 5-7). The items in each subscale were summed to a total score. For the negative experiences (score range 3-12), lower scores reflected more negative experiences with RPM. For the positive experiences (score range 3-12), higher scores reflected higher levels of more positive experiences with RPM. Item 1 was considered neutral and was calculated separately for each group.

The level of statistical significance was set to *P*<.05. The statistical analyses were conducted using SPSS Statistics for Windows, version 25.0 (IBM Corp).

The final open-ended question was analyzed with manifest qualitative content analysis [31]. The three authors who developed the survey and analyzed the qualitative data are researchers with years of experience as clinical nurses, two working in HF care (ML and AS) and one in ICD care (IT).

### Ethical Considerations

The study was approved by the Regional Ethics Committees for Human Research in Linköping, Sweden (ref 2017/441-31), and was conducted in accordance with the World Medical Association Declaration of Helsinki and the Code of Ethics for Nurses [32,33].

## Results

### Patients’ and ICD Nurses’ Characteristics

The sample consisted of 175 patients (response rate 98.9%) and 30 nurses (response rate 60%). The patients’ mean age was 69.9 years (SD 9.7); 138 out of 175 patients (78.9%) were males, 144 were retired (82.3%), and 109 were married (62.3%). A total of 128 out of 175 participants (86.0%) reported no symptoms of anxiety or depression, and 129 out of 175 patients (73.7%) scored a low level of ICD-related concerns (see Table 2).

The nurses’ mean age was 52.7 years (SD 8.7) and 26 out of 30 (86%) were females. They had been working as nurses for a mean of 26 years (range 5-47) and as ICD nurses for 7.6 years (range 1-14). They spent a mean of 7.5 hours/week (range 1-30) working with RPM (see Table 2).

**Table 2:** Characteristics of participating patients and nurses.

Characteristics		Value, n (%) or mean (SD)
**Gender (male), n (%)**		
	Patients (N=175)	138 (78.9)
	Nurses (N=30)	4 (13)
**Age (years), mean (SD)**		
	Patients (N=175)	69.9 (9.7)
	Nurses (N=30)	52.7 (8.7)
**Patient origin of birth (N=175),** **n (%)**		
	Sweden	153 (87.4)
	Other Nordic country	17 (9.7)
	Other part of Europe	5 (2.9)
**Patient living conditions (N=175), n (%)**		
	Single	57 (32.6)
	Married	109 (62.3)
	Living with spouse and child	7 (4.0)
	Living with relative	2 (1.2)
**Patient education (N=175), n (%)**		
	Elementary school	90 (51.4)
	Education after elementary school	63 (36.0)
	University or higher education	22 (12.6)
**Patient main occupation (N=175), n (%)**		
	Employed	18 (10.3)
	Self-employed	9 (5.1)
	Retired	144 (82.3)
	Sick leave	4 (2.3)
**Patient HADS^a^ score, anxiety (N=175)**		
	Total score, mean (SD)	3.6 (3.7)
	No symptoms (0-7), n (%)	128 (86.0)
	Mild symptoms (8-10), n (%)	10 (7.0)
	Moderate-to-severe symptoms (>10), n (%)	10 (7.0)
**Patient HADS score, depression (N=175)**		
	Total score, mean (SD)	3.6 (3.9)
	No symptoms (0-7), n (%)	124 (84.4)
	Mild symptoms (8-10), n (%)	19 (12.4)
	Moderate-to-severe symptoms (>10), n (%)	4 (2.8)
Patient CAS^b^ total score, mean (SD)		19.3 (5.2)
**Patient 8-item ICDC^c^ score (N=175)**		
	Total score, mean (SD)	6.3 (6.6)
	Low level of ICD^d^ concerns (0-10), n (%)	129 (73.7)
	High level of ICD concerns (11-28), n (%)	46 (26.3)
**Nurses only (N=30), mean (SD)**		
	Years since nurse exam	26.0 (9.4)
	Years working at device clinic	7.6 (3.7)
	ICD patients at the clinic	345 (205)
	ICD patients on remote monitoring	233 (290)
	Hours/week working with remote monitoring	7.5 (4.4)

^a^HADS: Hospital Anxiety and Depression Scale.

^b^CAS: Control Attitude Scale.

^c^ICDC: Patient Implantable Cardioverter-Defibrillator Concerns Questionnaire.

^d^ICD: implantable cardioverter-defibrillator.

### Experiences of Remote Monitoring

The majority of patients (154/175, 88.0%) as well as nurses (23/30, 77%) experienced RPM in general as very good. The mean scores of the negative experiences subscale were 11.5 (SD 1.1) for the patients and 10.7 (SD 0.9) for the nurses (*P=*.08), with a trend for the nurses being more negative than for the patients, although this was not statistically significant.

**Figure 1:**
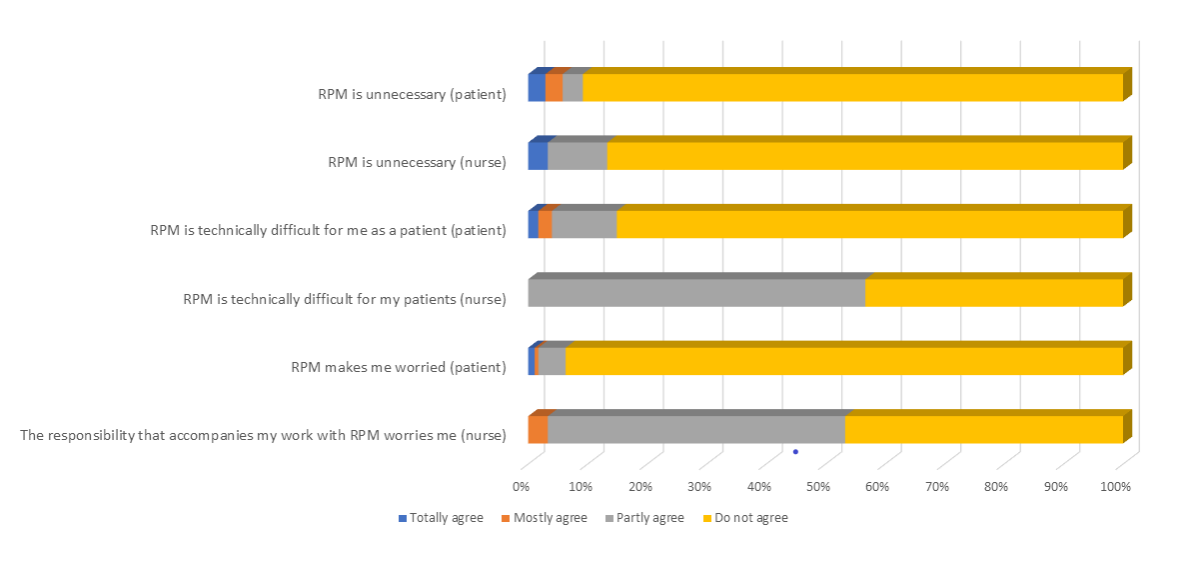
Negative experiences regarding remote patient monitoring (RPM) perceived by patients and nurses.

A total of 16 out of 175 patients (9.1%) and 4 out of 30 nurses (13%) found RPM unnecessary or partly unnecessary (see Figure 1). A total of 17 out of 30 nurses (57%) found the technical equipment somewhat difficult for the patients to handle; in contrast, 149 of the 175 patients (85.1%) answered that they did not experience any technical difficulties in handling RPM (*P=*.04). Only 11 out of 175 patients (6.3%) were worried or anxious about what the RPM entailed, while 15 out of 30 nurses (50%) felt distressed by the responsibility that accompanied their work with RPM (*P=*.04).

The mean scores of the positive experiences subscale were 11.1 (SD 1.6) for the patients and 8.5 (SD 1.9) for the nurses (*P=*.03), meaning that patients were more positive toward RPM than were the nurses.

**Figure 2:**
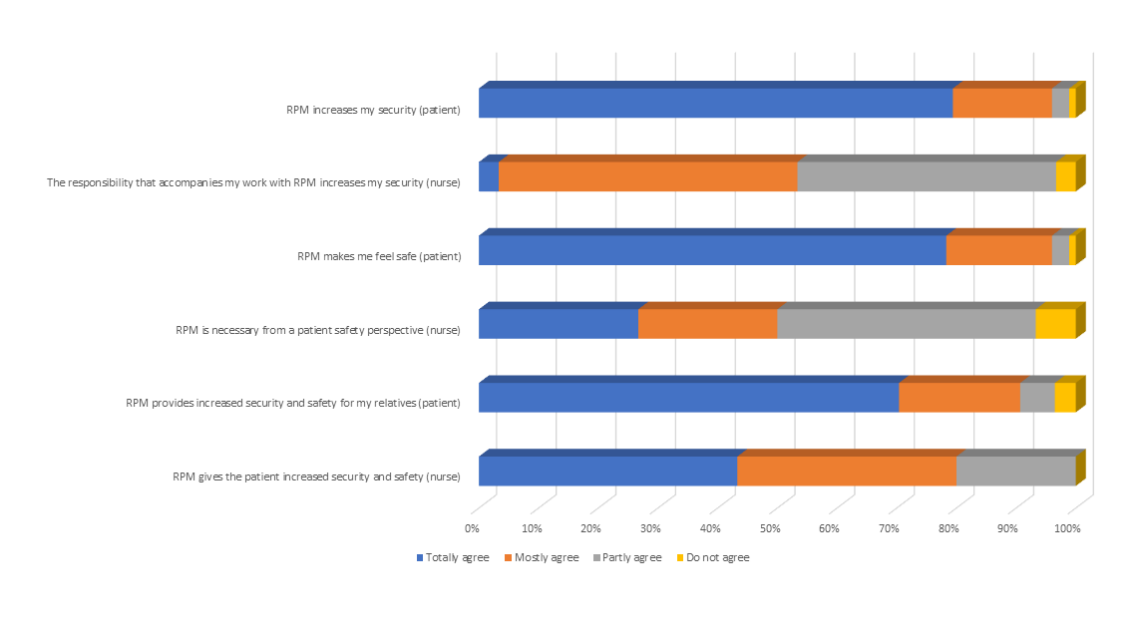
Positive experiences regarding remote patient monitoring (RPM) perceived by patients and nurses.

The majority of the patients found that RPM increased their own (173/175, 98.9%) and their relatives’ (169/175, 96.6%) security. All nurses (30/30, 100%) answered that RPM increased patient security and safety, and 28 out of 30 nurses (93%) found it necessary from a patient safety perspective (see Figure 2). Also, 15 out of 30 nurses (50%) answered that the responsibility that accompanied working with RPM increases their security, since they knew there would be an alert in case of malfunction or arrhythmias.

### Patients’ Perceptions About Being Monitored With RPM

#### Overview

In total, 101 out of 175 patients (57.7%) provided responses to the open-ended question regarding perceived advantages and disadvantages with RPM. The analysis resulted in 110 meaningful units, whereof 94 were described as advantages and 16 as disadvantages, which were further analyzed and categorized into two categories: *security and safety* and *organization of care*. Each category reflects both advantages and disadvantages with RPM.

#### Security and Safety

The most prominent advantage described was that RPM increased the sense of security and safety, not only for the patient but also for their relatives. Nevertheless, some patients highlighted the fact that atrial fibrillation was not automatically reported by one specific RPM manufacturer, which affected the feeling of security. Several patients expressed that they now could live a more “normal daily life,” not having to think about the ICD when knowing that someone was watching over them. However, misconceptions were also described where the patients believed that they were continuously monitored in real time, 24/7. Likewise, it was considered as a lack of safety when traveling and not bringing the home monitor, when patients were used to be monitored, which could cause worries.

#### Organization of Care

When it comes to the new way of organizing care, most of the patients described that it was positive not needing to travel to hospital for follow-up as often as before. In contrast, for some patients, the fewer number of office-based visits to the ICD clinic was considered a disadvantage since they appreciated the face-to-face interaction. The patients felt that they had no one to talk to when worries or questions arose and did not want to bother the nurse with a phone call. Still, others appreciated the possibility to send data to the ICD clinic, and it was described as reassuring to have the possibility to call the ICD nurse when needed. Finally, some patients emphasized the size of the home monitor and wished it to be smaller, while others reported that the control light shined too brightly during the night.

### ICD Nurses’ Perceptions About Working With RPM

#### Overview

A total of 23 out of 30 nurses (77%) responded to the open-ended question; this resulted in 76 meaningful units, whereof 31 were described as advantages and 45 as disadvantages, which were further analyzed and categorized into three categories: *security and safety*, *organization of care*, and *managing technology*. Each category reflects both advantages and disadvantages with RPM.

#### Security and Safety

In accordance with the patients’ perceptions, the nurses also described that RPM increased the safety and sense of security for the patients with early detection of arrhythmias, device malfunctions, low battery status, and decompensation of HF status. Early detection led to more immediate actions, and some alerts could be handled by phone instead of through an office-based visit. However, it was considered difficult to customize the correct alert limits for the individual patient, which often resulted in keeping the default alerts. This caused a high, and sometimes unnecessary, number of transmissions with a risk to miss severe arrhythmias and/or malfunction in the high flow of information.

#### Organization of Care

The nurses described that fewer office-based visits were an advantage for both the health care personnel and the patients. Fewer regular visits made it possible to schedule patients with short notice when needed. Nevertheless, the nurses also found the task assignments surrounding RPM, including all documentation in the medical records, to be burdensome. Some nurses stated that handling the transmission with interpretation of data, getting in contact with the patient in case of arrhythmia or device malfunction, consulting a physician if necessary, and finally documenting the alert took the same amount of time or more compared to an office-based visit. Some hospitals had not planned ahead before implementing RPM and, therefore, had no routines on how to document transmissions and lacked action plans on how to handle alerts. In these cases, RPM caused stress for the nurses, since they did not have any time set aside to handle all transmissions; they were then worried about missing important information. It was also described that hospital managers and heads of departments did not recognize the time-consuming work nurses did when handling daily transmissions. They just noticed that there were fewer patients at the clinic and tried to give the nurses other work tasks instead, which led to frustration and dissatisfaction.

#### Managing Technology

The nurses also described that having to learn all the different systems with different platforms and log-ins was difficult and stressful, especially for smaller clinics with few ICD patients, and the nurses wished for a joint platform for all manufacturers. They also highlighted how time-consuming it was to trace and handle different technical problems and the time it takes trying to reach the patient by phone. Extra stress was caused by all the time it took trying to reach the patient when the monitor lost contact with the server, which was described as a common technical problem. When there is no contact, patients experience a false sense of security, thinking that the nurses know about arrythmias when they do not.

### Patients’ and Nurses’ Weighted Results

Based on patients’ and nurses’ experiences of RPM, Table 3 presents weighted results with clinical implications and needed interventions for changed practice.

**Table 3:** Weighted results based on patients’ and nurses’ experiences of remote patient monitoring (RPM).

Weighted results		Clinical implications	Needed interventions for changed practice
**Experiences and satisfaction**			
	Both patients and nurses had good experiences of RPM, but patients were more positive than the nurses.	Acknowledge dissatisfaction among nurses and identify obstacles to work with RPM.	Engage device manufactures to arrange online seminars and support for nurses involved in RPM.
	A few patients and nurses found RPM unnecessary.	Continue to offer RPM to patients with implantable cardioverter-defibrillator (ICD).	Provide education and motivational support for RPM to every patient that receives an ICD.
	Some nurses found it challenging to customize the correct alert settings for the individual patient, which resulted in a high number of transmissions.	Provide practical information to nurses about how and when to individualize alert settings.	Engage device manufacturers to arrange online seminars and support for nurses involved in RPM.
**Security and safety**			
	Most patients experienced that RPM increased security, and this was in line with the nurses’ perceptions, since they knew there would be an alert in case of malfunction or arrhythmias.	Provide targeted written information about RPM to patients and nurses and highlight the security aspect.	Distribute a pamphlet with appropriate local information to patients and nurses new in RPM positions, in addition to the specific information from the manufacturer.
	Some patients highlighted that atrial fibrillation was not automatically reported by one specific RPM manufacturer, which affected the feeling of security.	Provide targeted written information about RPM to patients and give information about the data collection.	Distribute a pamphlet with information from the specific manufacturer and inform the patient about the possibility to perform a patient-initiated transmission in case of tachycardia.
**Technical aspects**			
	Nurses found the technical equipment difficult for the patients to handle more often than did the patients.	Identify the patient’s perceptions about how the RPM operates.	Offer technical support given by the manufacturer and provide the patients with written contact information. Arrange for timely and repeated group information targeting technical issues for patients.
	Some patients had misconceptions about being continuously monitored in real time, 24/7.	Identify the patient’s perceptions about how the RPM operates.	Proactively bring up how and when the data are being transferred to the clinic and the importance of calling the emergency service center in case of a life-threatening illness.
	Having to learn all the different systems with different platforms and log-ins was difficult and stressful for nurses.	Offer nurses new in the RPM position a mentorship program covering technical aspects and solutions.	Engage device manufacturers to arrange online seminars and support for nurses involved in RPM. Ask device manufacturers to help set up networks with nurses working with the same platform.
	Nurses highlighted how time-consuming it was to trace and handle different technical problems and the time it takes trying to reach the patient by phone (ie, when the monitor lost contact with the server).	Provide targeted written information about RPM to patients and nurses and highlight the technical aspects.	Offer technical support given by the manufacturer and provide the patients with written contact information. Arrange for timely and repeated group information targeting technical issues for patients.
**Emotional aspects**			
	Only few patients were worried or anxious about what the RPM entailed, while half of the nurses felt distressed by the responsibility that accompanied their work with RPM.	Identify the nurses’ perceptions about their workload when handling RPM.	Contact the device manufacturers and ask them to arrange online seminars and support for nurses involved in RPM. Deﬁne a written decision algorithm for the clinic in order to standardize the handling of transmissions.
	It was considered as a lack of safety by some patients when traveling and not bringing the home monitor.	Provide targeted written information about RPM and traveling routines to patients.	Distribute a pamphlet with appropriate local information, including a clear *travel plan* and how the individual patient is recommended to act during travel.
	Some patients felt that they had no one to talk to when questions arose and did not want to bother the nurse with a phone call, while others found it reassuring to have the possibility to call the ICD nurse when needed.	Acknowledge the emotional aspect of being an ICD recipient and identify those in need of extended support.	Proactively bring up the emotional aspect and offer emotional support. Provide written contact information for the clinic.
**Organization of care**			
	Most of the patients described that it was positive not needing to travel to hospital for follow-up as often as before, but some patients considered the fewer number of office-based visits to the ICD clinic as a disadvantage, since they appreciated the face-to-face interaction.	Identify the patient’s needs and wishes for follow-up of the device.	Offer person-centered care with individual follow-up appointments and/or provide telephone-based support in between the office-based follow-ups. Provide repeated patient education about RPM and its management using a person-centered approach and by applying, for example, teach-back methodology.
	Patients wanted to receive information directly from their remote monitored device.	Use automated direct call messaging for follow-up in patients with a device. Encourage patients to log in to their medical record electronically (when appropriate) to access the notes from the latest remote follow-up.	Using specific apps in smartphones, patients may have the possibility to check the website with information about their own device and to communicate or chat online with the health care personnel involved in the care of the patient in the future.
	Some hospitals had not planned ahead before implementing RPM; they had no routines on how to document transmissions and lacked action plans on how to handle alerts.	Identify the workflow and perform meticulous care planning before the implementation of RPM.	Deﬁne a written decision algorithm for the clinic in order to standardize the handling of transmissions.
	Hospital managers and heads of departments did not recognize the time-consuming work nurses did when handling daily transmissions.	Acknowledge nurses’ workloads by giving the heads of departments insight into how the remote transmissions impact on the regular appointments.	Provide a supportive environment for RPM (ie, activities that do not involve direct patient interaction), since the most frequently reported barrier for not implementing RPM is found to be lack of reimbursement. To prevent dissatisfaction by the nurses, new working structures might be necessary.

## Discussion

### Principal Findings

A main finding in this study was that both patients and nurses found several positive aspects with RPM, but the nurses noted more downsides than did the patients. There could be several explanations to this. Two survey studies exploring telemonitoring in HF in various countries found that health care professionals described several patient-related barriers due to physical or mental conditions. Further, the nurses have seen a range of different technical problems in different patients over time, while the individual patients only have their own experiences and, therefore, a more optimistic and positive view on the technology [34,35].

Patients expressed that they could live a more normal life after receiving RPM. This confirms results in previous research showing that patients were content with RPM and did not feel like patients as much anymore [16,36]. This is probably related to the fact that patients felt that RPM increased security and safety for both themselves and their relatives. Also, for the vast majority, it was considered as an advantage that RPM lead to fewer office-based visits. The advantage that RPM is less time-consuming than in-hospital follow-ups has also been seen in other studies [16]; however, both in this study and in previous research, the lack of direct face-to-face contact was missed by some patients [37].

Even as patients received information and education about RPM, some patients believed that RPM included live transmissions, both day and night, and that someone continuously watched their electrocardiograms as when being monitored with telemetry during a hospital stay. Ottenberg et al found that when patients were prescribed RPM for their ICD but then did not install the home monitor, it was largely attributed to not understanding the purpose of the RPM system or being unsure whether their system was correctly transmitting information [37]. This highlights the need for repeated patient education about RPM and its management using a person-centered approach and by applying, for example, teach-back methodology [38]. High-quality training has been found to improve patients’ understanding and comprehension and has been positively associated with anxiety and acceptance levels [23].

Downsides described by the nurses were often related to organizational issues; for example, a clear description of their own responsibilities handling alerts. Further, about half of the nurses also found the responsibility associated with managing RPM stressful due to different technologies, and they had concerns related to patient safety. A recent review describes the requirement of the referring nurse to be an expert in cardiac pacing and device follow-up, and a daily connection with a website should be performed to evaluate received alerts. To avoid stress and worries for the nurses, every center must deﬁne a written decision algorithm in order to standardize the handling of alerts [13].

It was also referred to as “invisible work” that is not recognized as time-consuming by managers and without proper reimbursement systems. It is important that health systems provide a supportive environment for RPM (ie, activities that do not involve direct patient interaction), since the most frequently reported barrier for not implementing RPM was found to be lack of reimbursement [17,39]. In the eHealth era, with increasing remote monitoring of various treatments, symptoms, and devices, adapting the organization of care is key and organizations need to adapt to best make use of remote monitoring.

Nurses also found handling the daily transmissions as burdensome and time-consuming since it was difficult to tailor the alarm limits for each patient. It is known, as shown previously, that many transmissions are patient initiated without any event. In a recent study by Ninni et al [40] that included 1423 transmissions, it was found that as many as 77% were initiated by the patients, and only about 3% of the transmissions per patient led to actions or interventions by the health care personnel. The authors stressed the need to optimize automatic transmissions and focus on patient education to reduce the workload at the device clinic.

In addition, technical concerns took a lot of time, with nurses trying to locate the problem and get in contact with the patient. This led to stress and an increased workload, and nurses also felt that it was a false safety for the patient who did not always realize that the transmission failed. A recent study highlights the encounter through telephone calls that took place related to home monitoring. Five types of clinical work were performed that may also refer to RPM: inclusion work, coordination work, diagnostic work, education work, and comfort work [41]. The authors found that telephone calls increased time spent in telemonitoring, and most telephone calls contained more than two issues [41].

Currently, patients do not receive information directly from their remote monitored device, which patients in this study pointed out as a downside. However, findings from a feasibility [42] and evaluation [20] study in the United States suggest that it is not only feasible to deliver data from remote monitoring directly to patients, but also that this data sharing does not adversely impact clinic workflow and that patients perceive a benefit from having access to their remote monitoring data. The same result was found by Mirro et al when evaluating the impact of sharing ICD data summaries through a patient portal. At the end of the study, two-thirds of patients were satisfied with the amount of information received through the electronic or paper ICD data summary. Further, providing patients with their device data did not increase ICD-specific clinical workload [43].

In the future, by using specific apps in smartphones, patients may have the ability to check the website with information about their own devices and to communicate or chat online with the health care personnel involved in the care of the patient.

### Conclusions

Both patients and ICD nurses found RPM to be safe and to increase the sense of security for patients and caregivers. There was a discrepancy between nurses and patients with regard to the technical equipment, where very few patients, but every other nurse, stated that the technology was difficult for the patients to handle.

Few patients were worried or anxious about the RPM, while half of the nurses felt distressed by the responsibility that accompanied their work. Nurses also described it as time-consuming to contact patients in case of alerts. To improve RPM from the perspective of the nurses, the organizational routines, reimbursement systems, and the balance of responsibilities and workloads need to be reviewed.

## Abbreviations

CRT: cardiac resynchronization therapy
CRT-D: cardiac resynchronization therapy defibrillator
EVOLVO: Evolution of Management Strategies of Heart Failure Patients With Implantable Defibrillators
HF: heart failure
ICD: implantable cardioverter-defibrillator
PREDICT-RM: Patient-Related Determinants of ICD Remote Monitoring
REMOTE-CIED: Remote patient management for Cardiac Implantable Electronic Devices
RPM: remote patient monitoring
